# The role, impact, and support of informal caregivers in the delivery of palliative care for patients with advanced cancer: A multi-country qualitative study

**DOI:** 10.1177/0269216320974925

**Published:** 2020-12-23

**Authors:** Samuel Ojima Adejoh, Florien Boele, David Akeju, Adlight Dandadzi, Elizabeth Nabirye, Eve Namisango, Elizabeth Namukwaya, Bassey Ebenso, Kennedy Nkhoma, Matthew J Allsop

**Affiliations:** 1 Department of Social Work, University of Lagos, Lagos, Nigeria; 2 Academic Unit of Health Economics, Leeds Institute of Health Sciences, University of Leeds, Leeds, UK; 3 Patient-Centred Outcomes Group, Leeds Institute of Medical Research at St James’s, St James’s University Hospital, Leeds, UK; 4 Department of Sociology, University of Lagos, Lagos, Nigeria; 5 Clinical Trials Research Centre, College of Health Sciences, University of Zimbabwe, Harare, Zimbabwe; 6 Department of Internal Medicine, Makerere University, Kampala, Uganda; 7 African Palliative Care Association, Kampala, Uganda; 8 Nuffield Centre for International Health and Development, University of Leeds, Leeds, UK; 9 Florence Nightingale Faculty of Nursing Midwifery and Palliative Care, Cicely Saunders Institute, King’s College London, London, UK; 10 Academic Unit of Palliative Care, Leeds Institute of Health Sciences, University of Leeds, Leeds, UK

**Keywords:** Palliative care, caregivers, neoplasms, Africa South of the Sahara, qualitative research

## Abstract

**Background:**

Cancer is increasing in its prevalence in sub-Saharan Africa. Informal caregivers are key to supporting engagement and interaction with palliative care services, but limited literature on their role impedes development of supportive interventions.

**Aim:**

We aimed to understand the role, impact, and support of informal caregivers of patients with advanced cancer when interacting with palliative care services in Nigeria, Uganda, and Zimbabwe.

**Design:**

Secondary analysis of qualitative interview transcripts. The dataset was assessed for fit and relevance and framework approach was used.

**Setting/participants:**

Interview transcripts of informal caregivers included participants aged over 18 years of age recruited from palliative care services across participating countries.

**Results:**

A total of 48 transcripts were analyzed. Mean age was 37 (range 19–75) with equal numbers of men and women. Five themes emerged from the data: (1) caregivers are coordinators of emotional, practical, and health service matters; (2) caregiving comes at a personal social and financial cost; (3) practical and emotional support received and required; (4) experience of interacting and liaising with palliative care services; and (5) barriers and recommendations relating to the involvement of palliative care.

**Conclusions:**

The role of informal caregivers is multi-faceted, with participants reporting taking care of the majority of medical, physical, financial, and emotional needs of the care recipient, often in the face of sacrifices relating to employment, finances, and their own health and social life. Efforts to develop comprehensive cancer control plans in sub-Saharan Africa must take account of the increasing evidence of informal caregiver needs.

**What is already known about the topic?**

- Informal caregivers are known to provide a crucial role in supporting patients with advanced cancer across multiple settings, but there is limited evidence of their role, impact, and support needs in the context of sub-Saharan Africa.
- It is necessary to understand the needs of informal caregivers in sub-Saharan Africa to guide augmentation and development of palliative care provision.
- Failure to adequately support informal caregivers could have an impact on advanced cancer patients’ supportive and palliative care.

**What this paper adds**

- We highlight the challenging role of caregiving for people with cancer, alongside context-bound features such as the financial, transport, and nutritional information needs of informal caregivers in sub-Saharan Africa.
- Informal caregivers take care of the majority of medical, physical, financial, and emotional needs of the patient, often incurring sacrifices relating to their employment, finances, health, and social life.
- A unique, balanced sample of male and female participants enabled us to explore potential gender differences in caregiver experiences and concerns, highlighting commonality in the overlaying multi-faceted high burden experienced by most participants.

**Implications for practice, theory or policy**

- Common needs of caregivers were identified across Nigeria, Uganda, and Zimbabwe which can guide efforts to augment palliative cancer care provision to support informal caregivers.
- The development of comprehensive cancer control programs in the participating countries should take account of the expressed support needs of informal caregivers highlighted in this study.
- Further research is needed to determine the mechanisms through which interventions could support informal caregivers of people with advanced cancer in sub-Saharan Africa and the subsequent employment, financial, and health sacrifices they experience.

## Introduction

Cancer is increasing in its prevalence globally, with a disproportionate rise projected in low- and middle-income countries.^1^ Specific to sub-Saharan Africa, in 2018, there were over 770,000 new cancer cases and 514,000 cancer-related deaths across Eastern, Middle, Southern and Western Africa.^2^ These figures are projected to rise further (up to 1.28 million new cases and 970,000 deaths) by 2030, with subsequent international and regional political declarations constituting a new global non-communicable disease agenda. In sub-Saharan Africa, approximately 80% of newly discovered cancers are no longer curable at the time of diagnosis. Access to basic palliative care and pain relief can be extremely limited,^3^ often due to late clinical presentation and poor access to facilities.^4^ Despite limited provision, palliative care remains the only realistic response to support equitable, accessible, and cost-effective interventions.

The delivery of holistic palliative care in sub-Saharan Africa includes three models; primary, secondary, and tertiary levels.^5,6^ Patients with advanced cancer commonly experience symptoms such as pain (87.5%), lack of energy (77.7%), drowsiness (72.3%), and worry (69.6%).^7^ The diagnosis and symptom burden can also create significant physical and emotional challenges for informal caregivers^8^—family or friends who provide patients with practical and emotional support. Informal caregivers can act as interpreters when patients have limited language skills^9^ and are often the main person managing patients’ symptoms.^10^ A previous systematic review on end-of-life care across multiple conditions in sub-Saharan Africa identified that informal caregivers are in practice, often providing the totality of care, without respite.^6^ Mostly women take on the caregiving role, usually without prior expertise, and many struggle to balance caregiving responsibilities with their everyday activities, family life, work, and social relationships.^11^

Positive consequences of informal caregiving include becoming more focused on priorities in the deeper sense of life and appreciation of life and support from other people.^12^ However, psychological distress is common in caregivers,^13–15^ and often severe caregiver burden spans across physical, psychological, financial, and social domains.^16^ Currently there is limited research in sub-Saharan Africa outlining the role of informal caregivers in the delivery of palliative care for advanced cancer patients, the impact of the caregiving role, and the types of support available to them.^6^ This impedes the development of interventions that may support informal caregivers. We seek to address this through exploring informal caregivers’ experiences of supporting people living with advanced cancer and understanding current interaction and support provided from palliative care across three countries: Nigeria, Uganda, and Zimbabwe.

## Methods

### Research question

What is the role, impact, and support of informal caregivers of patients with advanced cancer when interacting with palliative care services in Nigeria, Uganda, and Zimbabwe?

### Design and setting

This study emerged from a multi-country cross-sectional qualitative study in three countries in sub-Saharan Africa (Nigeria, Uganda, and Zimbabwe)^17^ to determine optimal mechanisms through which patient-level data can be used in the development and delivery of palliative cancer care. Following the main analysis of 195 stakeholder interviews from the parent study, we sought to further explore the rich and detailed data from caregiver interviews. Our rationale was to contribute to a very limited evidence base relating to informal caregivers for patients with advanced cancer in sub-Saharan Africa, gain knowledge of unmet support needs and inform future development of supportive interventions. Our qualitative secondary analysis^18^ sought to re-analyze data to bring new substantive insights and generate new knowledge on the role, impact, and experience of informal caregivers in interacting with palliative care services to support patients with advanced cancer.^19,20^ We assessed the caregiver transcripts and research question explored in this study against a rubric^21^ to provide assurance of fit and relevance of pre-existing qualitative data to this secondary analysis.

Caregiver participants had been purposively sampled for face-to-face interviews from nine palliative care provider sites across participating countries,^17^ with country selection informed by consultation with the lead project partner, the African Palliative Care Association. Recruitment occurred across primary, secondary, and tertiary facilities in each country. Eligible participants included caregivers who were at least 18 years of age and identified by the patient as: “*unpaid, informal providers of one or more physical, social, practical, and emotional tasks. . . they may be a friend, partner, ex-partner, sibling, parent, child or other blood or non-blood relative.*”^22^ Half of the participants were caregivers of patients who also participated in the parent study, and half participated independently. The sample size was based on previous experience of the research team^23,24^ and research literature.^25,26^ We estimated that recruitment of 15 caregivers per country would achieve data saturation, with ongoing analysis to monitor emergence of new themes during conduct of interviews, with a target sample of 45 caregivers overall. The sampling criteria (sex and age) were informed by previous differences identified in the experiences of caregivers.^27^

Clinical leads in each country liaised with palliative care providers to identify eligible participants, confirming they were aware of a patient’s cancer and that palliative care was being delivered. Caregivers were introduced to a researcher and provided with study information with a week to consider participation. Semi-structured interviews were used to enable reciprocity between the interviewer and participant, allowing further exploration or follow-up by interviewers and providing flexibility in discussions about potentially upsetting and sensitive topics. Interviews took place with individual interviewees on their own at home or in a private location in a clinic setting and were audio-recorded. Topic guides were framed around the experience of being a caregiver for a person with advanced cancer, current interaction with and access to palliative care services, and anticipated clinical responses from health services (whether communicating in person or via digital technologies). The topic guide was pilot tested via teleconference by researchers involved in data collection to refine the content and support familiarization with the flow of questioning. Researchers included university lecturers (SA, DA), health researchers (AD; ENab; ENami), and palliative care professionals with doctoral training in health research (ENamu). Interviewers were closely supported through regular communication with experienced and global health researchers (MJA, KN, BE). All interviewers were fluent in English and the local languages (Yoruba in Nigeria, Luganda in Uganda, and Shona in Zimbabwe). Interviewers were flexible and fluid in which language was spoken. After each interview, researcher notes were written, including key points arising from discussions and difficulties experienced. Interviews were conducted between February and August in 2019.

### Data analysis

All interviews were transcribed verbatim and back-translated into English where necessary (supported by an independent translator) before being imported into NVivo version 12. Adopting the framework approach to thematic analysis,^28^ we focused on caregiver experiences supporting patients with advanced cancer in interacting with palliative care services. Two postdoctoral health researchers with experience in cancer research, a male (SOA) and a female (FB), read and reread the transcripts. One of the authors (SOA) had conducted interviews across all stakeholder groups in Nigeria so benefitted from an in-depth knowledge of the parent study. Six transcripts were randomly selected across the three countries, from where the initial categories and subcategories were created and defined by two authors independently (SOA, FB). The two authors (SOA, FB) then met to compare the similarities and differences and to agree on the categories to be used to continue coding, supported by a third author (MJA) as needed. This process was repeated per six transcripts until all transcripts were coded, expanding the coding framework with new categories as needed, and regular discussions leading to consensus. Although nuances within sub-themes were still emerging toward the end of data analysis, the themes were being replicated, indicating a level of completeness across countries and sample characteristics. We reported findings along the themes and subthemes identified and in alignment with the Consolidated criteria for reporting qualitative research (COREQ).^29^

## Findings

Qualitative semi-structured interviews were conducted with 48 participants from across three countries, representing each level of palliative care delivery (primary, secondary, tertiary). One participant declined to participate as they were unavailable during the interview period. The mean age was 37 years old (ranging from 19 to 75 years), with an equal number of male and female caregivers. Most participants had attained secondary (45.83%) or tertiary (41.67%) education and the majority were either married (47.92%) or single (45.83%). Demographic characteristics of participants are outlined in Table 1. Interviews ranged from 17 to 70 min and lasted on average 50 min. The shorter duration is accounted for by two caregivers who were new to caregiving with limited experience of caregiving and its impact.

**Table 1.** Overview of participant characteristics.

Participants (*n* = 48)	
Mean age (SD)	37 (13.44)
Sex	
Male	24 (50%)
Female	24 (50%)
Country	
Nigeria	18 (37.50%)
Uganda	15 (31.25%)
Zimbabwe	15 (31.25%)
Education	
Secondary	22 (45.83%)
Tertiary	20 (41.67%)
Primary	4 (8.33%)
No education	2 (4.17%)
Marital status	
Married	23 (47.92%)
Single	22 (45.83%)
Widow	3 (6.25%)
Religion	
Pentecostal	18 (37.50%)
Roman Catholic	12 (25.00%)
Anglican	4 (8.33%)
Apostolic	4 (8.33%)
Methodist	2 (4.17%)
Jehovah witness	2 (4.17%)
No religion	6 (12.50%)
Carer relationship	
Sibling	18 (37.50%)
Son/daughter	15 (31.25%)
Husband	7 (14.58%)
Parent	5 (8.33%)
Wife	4 (8.33%)

**Figure 1.**
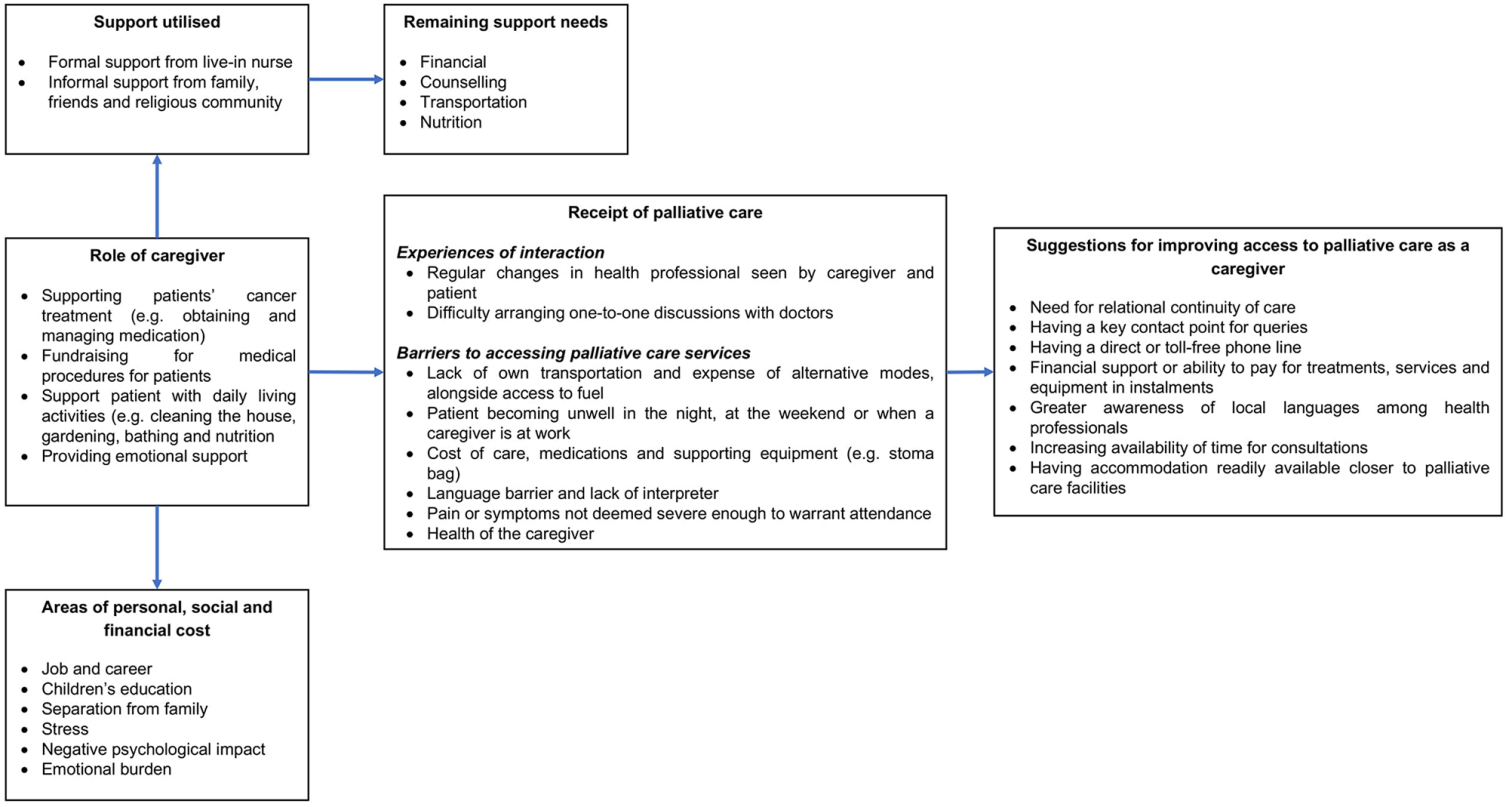
Overview of key themes arising from the analysis of caregiver interviews.

Five themes emerged, as summarized in Figure 1 and outlined below.

### Caregivers are coordinators of emotional, practical, and health service matters

Caregivers support patients with advanced cancer in numerous ways without clear differences between Uganda, Nigeria, and Zimbabwe. Many caregivers, irrespective of age or sex, mention actively supporting patients’ cancer treatment. This includes obtaining and managing medications.

*“I take care of her. . . just like I went to the store to help her there, then after I come back from the store I still help her at home. Whenever she is feeling pain I will ask her what is wrong with you she will tell me she is feeling pain, then I will ask her to take drugs so that the pain will come down. Sometimes she will tell me that no, I will still plead with her that she should take the drugs, she will take the drugs, sometimes she will be vomiting, I will still run to her, give her water I will be telling her sorry, sorry so that she will feel better and be happy.”* Female, 22, Nigeria

Caregivers actively interact with and manage communication with services, making efforts to make and attend appointments in the clinic, sometimes attending on behalf of the patient if they were physically or practically unable to travel.

*“Yes, we take a lot of time seated in the sitting room, then you wait for the receptionists to call you and you go through the process you end up to leave late, and when you are seated there your heart is divided. You know what you have left behind, the person is in terrible pain or maybe I wish I could get oral morphine something I have come for and leave this place.”* Male, 39, Uganda

This is interconnected with finances and fundraising for medical procedures, which is difficult for many and particularly raised by caregivers from Uganda and Zimbabwe.

*“In the process of talking, examining the issue and discussing with others, some people said, why don’t you go to Mesina [town in South Africa close to Zimbabwe] and have MRI done there. It is cheap. Cheaper than. . . here in Zimbabwe. I then said let me find out. Then I, I still had a car at that time. The father had left a car behind. People from the church eh, from <local church denomination> then contributed about 700 dollars which they had pledged to give. We refueled the car with petrol and then went to South Africa.”* Female, 46, Zimbabwe

At home, caregivers support patients with (instrumental) activities of daily living, ranging from maintaining the garden, cleaning the house, and doing laundry to more basic activities of daily living like providing nutrition and bathing. Nutrition in particular is mentioned frequently in all three countries, linked with common beliefs that food has a role in improving health, seeming to provide caregivers with some sense of control over the situation.

*“So, we studied that when it comes to the issue of food, when a person is diagnosed with the cancer disease (. . .) it destroys body cells and other things. So, we need to find food which increases a person’s cells so that we can fight the. . . let us suppose that there are 10 cells which are being destroyed per day. If we give them food which increases the cells, that will ensure that the life of that person is lengthened somewhat due to the fact that we would be ensuring that for every cell destroyed, we replace with other cells. For each cell lost, we replace with other cells. That might help.”* Male, 19, Zimbabwe

Furthermore, many caregivers mention the importance of providing emotional support to the patient as the disease and treatment could weigh heavily on patients’ mental health.

*“Like psychologically it has weighed him down, he tends to think almost every time whenever he is alone, he is in his room he thinks a lot that is the major reason we don’t like leaving him alone because we know he will start thinking, start feeling somehow that is why we to play with him, check on him.”* Male, 24, Nigeria

### Caregiving comes at a personal, social, and financial cost

Across Uganda, Nigeria, and Zimbabwe, juggling care responsibilities with their own everyday activities, family life, and work takes a toll and many caregivers have to make significant changes to their lives. This includes giving up their job or passing on career opportunities to provide care, reported by both male and female participants, particularly by younger caregivers.

*“I was working but now, ever since my mother fell sick, I am not working. At my work place they used to give me some days off but my return days would coincide with patient’s review dates. And my mother could not go for review alone, so I got confused of what to do. I decided to leave the job and take care of my mother, my patient. I did not have support and I was the only caregiver, I was married, I needed the job and also my mother needed my support, and I could not leave her alone.”* Female, 24, Uganda

This has repercussions on their financial situation with sometimes far-reaching consequences for the wider family. One caregiver describes the impact on their children’s education, as well as it leading to a period of homelessness.

*“The children, in fact, I have to withdraw the children from private school. They all going to public school just to in fact just to give myself little degree of mouth. (. . .) By 2016, I lost, we lost, we were not able to pay accommodation, we have to lodge for two years. The landlord take over, took possession of the house then we had to go to friends, my load as I speak is still spread outside. Then, err, a church member just allow her to stay at the balcony that’s where she has been. Rain she is there, sun she is there, night, mosquito everything, she is there. That way have tried to get accommodation, this January God provided a place for us, that we just move on the floor now, we are lying, just lie on the floor.”* Male, 62, Nigeria

In other cases, caregivers have had to move in with the patient in order to provide care, thereby causing “separation” from immediate family members of children and husbands, although a patient moving into a caregiver’s home was more commonplace in Zimbabwe. For many caregivers, providing care leads to stress and has a negative psychological impact. Some caregivers describe stigma associated with disease adding to this emotional burden.

*“Aaahm, cancer is a tough diagnosis (he laughs),it is a tough diagnosis, it drains you emotionally and also initially the hardest struggle for me was that element of trying to deal with the stigma, because she was actually a bit stigmatized. she didn’t even want close people to know, but when we went to hospice and we talked to the doctors there, we were advised and saw value in disclosure, and that was actually the hardest part of it, and also to accept later, to accept. you know she is also a medical worker and so understanding to transition from being a medical worker (he laughs) to now becoming a patient was what we also struggled with. But later on we were like whatever the doctor suggested, we shouldn’t be overriding. If they said we do this we should follow.”* Male, 36, Uganda

A minority of caregivers from Uganda however, also reported that providing care has led to a positive psychological impact, highlighting the role of faith and feeling thankful for the opportunity to care for their loved one.

*“I think it changed my life because there are circumstances that come and I think, we thank God, I thank God because of an opportunity to take care for her and I thank God for probably it is not good but also good coming to hospital and interacting with other patients and also working with the doctors and nurses also seeing this as a part of life! You know when everything is comfortable you may not get to know the other part of life; what people go through. Yah, I can say it’s been a blessing in disguise and also learning more about cancer.”* (Female, 44, Uganda)

### Practical and emotional support received and required

Some caregivers report receiving some formal support, such as a live-in nurse, or informal support from family, friends, and the (religious) community. This ranges from practical and emotional support including praying, to fundraising for medical treatment, alongside acquaintances with a medical background helping in the decision-making process. However, a frequently recurring theme across the three countries was the need for additional support for both patient and caregiver. Financial support was frequently mentioned, as was the need for counselling for both patient and caregiver to mitigate some of the psychological consequences of cancer.

*“I think they should focus on counselling more than anything else especially for the patients. Ah, as for the caregivers, yes, personally I’m also starting to think that. So, my mum, so, my mum, even all her other children begin to think about it but the issue. . .they are supposed to counsel the patient herself more. . . and physio [physiotherapy] could be done. A person might get to walk but it is counselling that is important.”* Female, 37, Zimbabwe

Apart from these areas, country-specific support needs identified by caregivers included help with transportation, nutrition, having another family member or friend to accompany the patient, and learning how to effectively communicate with palliative care (Zimbabwe); transportation and a toll-free phone line to communicate with palliative care (Uganda); and adequate attention from, and time with providers (Nigeria).

### Experience of interacting and liaising with palliative care services

Across the three countries, caregivers were currently in communication with palliative care supporting a patient with advanced cancer. In Uganda and Nigeria, caregivers expressed concerns related to interpersonal relationships and communication between caregivers and doctors.

*“Uhm, it has been somehow easy, much as. . . it may be quite different because for example, at hospice, I wish they could assign only one medical personnel to a patients, because it’s easy, it might be easy today I go to hospice I find maybe a male medical officer, next time see a different face and the other one will be so different face, so different faces sometimes becomes a little bit harder. Sometimes they ask what you went through, sometimes something is very distressing, for her to remember the history of the patient and all that, but it would be much better, it may be easier if a patient is assigned to one, knowing that am going to hospice, am going to find so and so.”* Male, 39, Uganda

In Uganda, caregivers described having easy access to health professionals but were worried about the continuity of care as they described often meeting different health professionals. Repeatedly explaining the patient’s condition caused caregivers to feel uncomfortable and distressed.

In Nigeria, caregivers expressed the challenges of arranging a one-on-one discussion with the doctors.

*“Is not that easy because once you are trying to share anything with them they are either walking away or attending to someone telling you to hold on, at last they won’t later have that time again, they will say they are in hurry or going to somewhere else, that so, there are so many times, they are so many attempts are being made even if you are trying to see them self in their various offices they might say they have somewhere to go, they have a meeting to attend to, I have something to attend to, so it has not been easy.”* Male, 28, Nigeria

Caregivers identified barriers to why they may not be able to access palliative care for their patients. These factors cut across the three countries studied. Time of day was linked to access, where support needs late at night or at weekends was difficult and doctors were often not available for contact. Transportation intricately linked with distance was mentioned by caregivers as a barrier to palliative care.

*“The problems I get are that at times transport can be a problem in our village, the vehicles are scarce and the roads are very bad, and also during the rainy season the roads get flooded and the vehicles can’t pass. . . yes, at times when she quite unwell and very weak, you can’t use public means like a taxi, you need private means, and it is very expensive, because the distance is very far.”* Female, 24, Uganda

Distance, bad roads, poor condition of the patient, and time of day (again, with night-time being especially difficult) compound transportation issues.

*“For me to go and collect pills at <<local Hospital>>, it’s something that is hard for us because sometimes there will be no money. . . For her to get out of the house and board an omnibus it’s hard, she would be in pain like when the omnibus is moving and she gets shook, the pain increases. So, using an omnibus is sort of hanging in there but she would be in pain.”* Female, 19, Zimbabwe

Cost is an important factor as many caregivers do not own private vehicles or cars, and renting or hiring a taxi is very expensive. Getting fuel can be a major problem, which was particularly problematic for those in Zimbabwe which has countrywide fuel shortages. Caregivers pointed out that money is one major barrier to accessing palliative care for their patients. This includes the cost of care, medications and necessities such as stoma bags, making phone calls, and transportation.

*“. . .. Ahhh maybe there are times when you don’t have that money that they ask for to get [stoma] bags. The contribution. . . because there is a fee that you have to pay to get the bags. You just forego and you wait until you get that money and then you go. . . like, you can’t do without the bags it’s a daily thing so we need them, if they are finished you really need them as soon as possible.”* Female, 31, Uganda

Some caregivers expressed the lack of information as the reason they did not access palliative care at a point in time, such as not being sure of working hours and availability on public holidays.

Unlike in Zimbabwe and Nigeria, caregivers in Uganda expressed difficulties in communicating with palliative care due to a language barrier and needing an interpreter.

*“It depends on the two parties say the doctor or. . . the patient may be a Lugbara [one of the tribes in Uganda], they don’t know English and the doctor doesn’t know Lugbara so there is language barrier. One explains things the other doesn’t understand; so you need an interpreter. . . yes.”* Male, 50, Uganda

Caregivers in Uganda could forego making an appointment if they judged that the patient’s pain is not severe enough, or if they are unable to confirm the availability of the health professionals.

*“Well, we’ve been given appointments so we could follow the appointments, but sometimes we fail to fulfil the appointments due to sometimes transport, and at times when we see there is really no necessity. if he has no. . .like, too much pain, or the condition is not bad, at times we find that we have to miss the appointment.”* Male, 52, Uganda

Caregivers in Uganda described clashes between their paid employment, and the timing of palliative care appointments. This caused them to forgo work in order to attend the palliative care appointment.

*“Another issue would be time. More especially when you are supposed to go, you are supposed to travel to hospice, you know you have also work to do. Now at times you try to manage time, and then say, maybe you are employed, then your employers need you at a certain time, and then hospice, by the time you will be leaving your work hospice will be closed.”* Male, 38, Uganda

Caregivers explained that feeling physically unwell themselves can cause a barrier to accessing palliative care for their patient.

*“There times also when you yourself the care giver you are unwell. . .There are times when you can also be unwell and you can’t, moving or travelling to the hospital you are not feeling fine.”* Female, 31, Uganda

### Recommendations relating to improving access to palliative care

To overcome some of the barriers outlined, caregivers from all three countries offered suggestions. These included the need for better continuity of care; being able to contact one key person; having a direct and toll-free phone line; financial support through, for example, government investment in palliative care or the option of paying for services in instalments; increased time for consultations; palliative care health professionals learning local languages to improve communication; and access to accommodation closer to palliative care when the patient is very ill.

*“If there is any way hospice can pass through the government and there is some fund they give to, from the government to hospice, because at times, these people, there are services they need and hospice cannot give. . . there are medicines they need that hospice do not have. And these people buy them. And these people, these cancer people, it takes. . .most of them are really broke – many of them, by the time you contact, or you go to hospice, you are really broke. . . financially broke or bankrupt, because as I told you before, that this is a sickness which is very expensive. Sometimes, because there are drugs, medicines which they get from hospice, but not that every medicine a cancer patient needs hospice has it.”* Male, 52, Uganda

*“Ummm, you need assist so we can communicate with the people [at the hospice]. To know how to communicate with them so that we can easily get hold of them and also on transport. To know how they can assist the patients who come to see with transport. If the patients get serious can they emergency for them or to organise something to come and get them it is better.”* Female, 38, Zimbabwe

*“. . .just the patient to be able to explain in details and their time not to be in a hurry and even if they are in a hurry to give us other time we can, we can actually reach them ok maybe, ha I am busy now I need to attend to 1 million patient, okay, but because you are, can call me at this time or you can call at this time then we will have enough time to talk to him. I think so just the patient to be able to explain in details and their time not to be in a hurry and even if they are in a hurry to give us other time we can, we can actually reach them ok maybe. . .”* Female, 43, Nigeria

## Discussion

### Main findings

This is one of the first studies to describe informal caregiver experiences and the impact of supporting people living with advanced cancer and facilitating access to palliative care in Nigeria, Uganda, and Zimbabwe. Multiple aspects of the role and impact of caregivers were identified which mostly cut across these three countries. Our study confirms that caregivers are a crucial partner in the provision of palliative care for advanced cancer patients, taking care of the majority of patients’ medical, physical, financial (e.g. fundraising for treatment), emotional, and spiritual (e.g. praying) needs. The caregiver burden across the three countries involves varying sacrifice relating to employment, finances, and their own health and social life. Previous research has outlined greater objective and subjective burden due to unpaid care work in low- and middle-income countries than in high-income countries.^30^ Indeed, there is increasing evidence of considerable and neglected burden on caregivers in low- and middle-income countries in physical, psychological, social, time, and financial realms.^31^ Our study provides new evidence of the many challenges faced by informal caregivers of people with advanced cancer, the impact on their lives, and the ways in which palliative care services do and could provide support. We add new perspectives from countries in sub-Saharan Africa to conclusions from a recent systematic review aimed at identifying the unmet care needs in patients with advanced cancer and their informal caregivers.^32^ Our results highlight the challenging role of caregiving, alongside context-bound features such as unmet financial, transport, and nutritional information needs. This work develops the evidence base on experiences of informal caregivers of people with cancer in sub-Saharan Africa, and is the first qualitative exploration of this population in Zimbabwe.

Study participants were family members with a mean age of 37 years, mirroring similar characteristics found for informal caregivers in other low resource settings.^33^ While research has begun to explore determinants of caregiver burden,^34^ very little research has been undertaken to determine specific caregiver needs in advanced cancer in the sub-Saharan Africa region. Participants in our study were balancing multiple priorities, often to their own detriment, worsened by poverty. Due to care responsibilities, work, family, and their own physical and psychological health were adversely impacted. Findings from this study align with a limited example of research from South Africa.^11^ Whilst a product of our recruitment strategy, the sample was unique with a balance of male and female participants allowing us to explore potential gender differences in caregiver experiences. Recent research suggests that, in the context of low- and middle-income countries, there are differences in the prominence and manifestation of caregiver burden between men and women.^35^ Although clear gender differences were not evident in our study, this may in part be because of the overlaying multi-faceted high burden experienced by most participants. In Africa, caregiving is seen as a predominantly female practice, though this may be changing.^36^ Further research on informal caregivers’ experiences within their relevant socio-cultural contexts is required to support the development of interventions that target evidence-based culturally appropriate structures.

We identified particularly pressing financial and transport needs. This aligns with experiences reported in other settings, where similarly almost all areas of caregiving are impacted by the financial status of the patient and family.^37^ The needs expressed by caregivers were very much linked with the burden; there was a pressing need for financial support and counselling. Approaches to developing supportive interventions for cancer caregivers are increasing, although an absence of high-quality studies makes it difficult to derive recommendations for practice.^38,39^ Previous research has focused on developing interventions for underserved caregiver populations. For example, in the US there are efforts to develop individualized goal-directed interventions to support caregiver self-care strategies for those faced with financial burden^40^ alongside efforts to guide care for caregivers in oncology settings in Canada.^39^ A clear need remains for similar evidence-based intervention development to support informal caregivers in sub-Saharan Africa.^41^ Our findings can directly inform such efforts, ensuring caregivers’ perspectives and their involvement are central to the design and implementation of supportive interventions,^42^ highlighting the multiple domains which are likely to require multidisciplinary team responses.^32^

Patients and informal caregivers can experience better continuity of care by interacting with a small number of trusted healthcare professionals, who provide multidisciplinary care and regularly transfer information to all healthcare professionals involved.^43^ Our study showed a clear unmet need for such a key worker. The need for greater continuity of care across the cancer care continuum in high income-countries has led to, for example, patient navigator services, facilitating linkages to follow-up services, and reducing or eliminating barriers to cancer care.^44^ Early studies suggest a similar approach may support improvements to patient outcomes in low- and middle-income countries^45^ although their feasibility, scalability, and sustainability in this context are unclear. Furthermore, there may be scope to circumvent practical barriers identified by our participants (distance, transport, costs) through developing approaches that leverage increasing access to digital technologies in the region.^46^ Increasing evidence suggests preliminary efficacy of digital interventions to improve patient-provider communication.^47^ Such augmentation of care may, for example, support caregivers in deciding whether or not to attend a clinic based on their perception of the patient’s symptom severity.

### Limitations of the study

This study was conducted concurrently in three sub-Saharan African countries with a large and diverse participant sample. Sampling criteria included both men and women, with half of each recruited to the study. The in-depth, rich data collection in the parent study enabled this secondary analysis focusing on solely caregiver perspectives. Limitations include that the parent study had a different primary aim, which means further elaboration and seeking of specific responses to address the focus of this secondary analysis was not possible. The concurrent activity across three countries meant that three different teams were collecting data. However, training was provided by the lead research team (MA, KN) and validation checks of transcripts took place regularly.

## Conclusion

Informal caregivers are a major partner in providing advanced cancer patients with palliative care in sub-Saharan Africa. This comparative, in-depth analysis of the role, impact, and support of informal caregivers of advanced cancer patients across three countries highlighted that they provide the majority of medical, physical, financial, and emotional needs of the patient, often incurring sacrifices relating to their employment, finances, health, and social life. We highlight novel caregiver experiences that are unique to the three participating countries, which includes an increased burden arising from a lack of access to transport, finances, and work. Further research is required to better explore how these findings can inform future adaptation of practice and intervention development targeting caregivers. This could be an important step toward improving palliative care pathways to better support caregivers in providing patients with the best possible palliative care at home.
